# Exercise Snacking in Alzheimer's Disease: A Mechanistic Rationale Based on Repeated Exerkine Signaling

**DOI:** 10.1111/jnc.70517

**Published:** 2026-07-04

**Authors:** Ricardo A. L. De Sousa, Joyce Mirlane Moreira Costa, Ramona Ramalho de Souza Pereira, Leiriel Cristina dos Reis Vieira, Caíque Olegário Diniz e Magalhães, Marco Fabrício Dias‐Peixoto

**Affiliations:** ^1^ Wright State University Dayton Ohio USA; ^2^ Postgraduate Program in Health Sciences Federal University of the Jequitinhonha and Mucuri Valleys (UFVJM) Diamantina Minas Gerais Brazil; ^3^ Department of Physical Education Federal University of the Jequitinhonha and Mucuri Valleys (UFVJM) Diamantina Minas Gerais Brazil

## Abstract

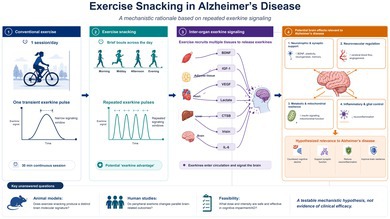

## Graphical abstract

Proposed mechanistic framework by which exercise snacking may provide repeated exerkine signaling windows relevant to Alzheimer’s disease. Compared with a single exercise session, brief exercise bouts distributed across the day may generate repeated exerkine pulses, promoting inter‐organ signaling from skeletal muscle and other metabolic tissues to the brain. This repeated signaling pattern is hypothesized to support neurotrophic and synaptic plasticity, neurovascular regulation, metabolic and mitochondrial resilience, and inflammatory/glial control. This model represents a testable mechanistic hypothesis and should not be interpreted as evidence of clinical efficacy.

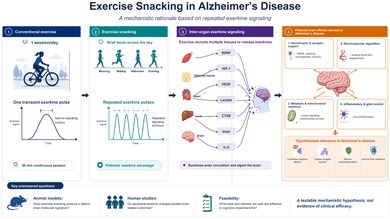

Exercise is one of the few non‐pharmacological strategies with the potential to affect several biological processes involved in Alzheimer's disease, including cognitive decline, neuroinflammation, cerebrovascular dysfunction, impaired insulin signaling, mitochondrial stress, and loss of synaptic plasticity (Pahlavani 2023). However, most discussions of exercise in patients with Alzheimer's disease still center on duration, intensity, or modality. A simpler question has received less attention: when are exercise‐induced molecular signals delivered?

This question is important because many molecular responses that may carry the benefits of exercise to the brain are transient. Circulating brain‐derived neurotrophic factor (BDNF), for instance, rises after moderate‐to‐vigorous exercise, but the increase is short‐lived, with concentrations returning to baseline within 15–60 min (Jemni et al. 2023). Therefore, a conventional once‐daily exercise session may only open a narrow window for molecular signaling.

Current guidelines recommend that adults accumulate at least 150 min/week of moderate‐intensity aerobic activity, often translated in practice into 30‐min sessions on most days of the week (Bull et al. 2020). The approach is sound in principle but hard to sustain in practice, especially for older adults contending with fatigue, low motivation, limited mobility, cognitive impairment, comorbidities, or fear of injury.

Exercise snacking (ES) refers to brief bouts of exercise, lasting from a few seconds to a few minutes, that are distributed across the day rather than concentrated in a single daily session. Although many ES protocols use vigorous or high‐intensity exercise, ES and HIIT are not synonymous (Weston et al. 2025).

Could ES offer a practical strategy for people living with Alzheimer's disease? ES has recently been proposed as an alternative for people with severe mental illness, in whom fatigue, low motivation, limited access, and poor adherence often restrict participation in conventional exercise programs (Trott et al. 2026). ES may provide a more feasible way for individuals with Alzheimer's disease to engage in regular exercise. However, a key issue for Alzheimer's disease is not only the feasibility of ES. In our most recent editorial on ES for surgical prehabilitation in cancer, we used the term “the exerkine advantage” (Dias‐Peixoto et al. 2026). Exerkines are a diverse group of humoral factors released by multiple tissues during or after acute exercise (Chow et al. 2022). Importantly, exerkines differ substantially in their release kinetics, tissue sources, transport mechanisms, and downstream signaling; repeated exercise bouts are therefore unlikely to affect all candidate mediators in the same manner. In our recent article, we proposed a simple premise: several short bouts of exercise may expose tissues to repeated, transient surges of exerkines rather than concentrating the molecular response in a single post‐exercise window (Dias‐Peixoto et al. 2026). We now ask whether the same temporal principle applies to Alzheimer's disease. The argument is not that exercise snacking merely increases physical activity. By distributing exercise throughout the day, the timing of exercise‐derived signals, their recurrence, and whether the brain encounters them as isolated events or as a repeated signaling pattern may be altered. However, this temporal framework is derived mainly from acute exercise studies and preclinical models. Whether ES produces repeated exerkine responses in Alzheimer's disease, and whether these signals reach or influence the brain and modify disease‐relevant outcomes, has not been directly established. Accordingly, the framework proposed here should be viewed as a testable mechanistic hypothesis rather than evidence of clinical efficacy.

Candidate exerkine molecules that act in the brain include BDNF, IGF‐1, VEGF, lactate, cathepsin B (CTSB), irisin, and interleukin‐6 (IL‐6). Muscles are central to this response, but they are not alone. Exercise also recruits adipose tissue, heart, liver, and the brain itself, turning a local contraction into a wider organ‐to‐brain conversation (Figure 1). In Alzheimer's disease, this inter‐organ signaling may be particularly important to four interconnected processes: neurotrophic and synaptic support, neurovascular regulation, metabolic and mitochondrial resilience, and inflammatory and glial control. Of the four, the neurotrophic and synaptic link is the most immediate, and BDNF is its central mediator. It supports neuronal survival, synaptic plasticity, hippocampal neurogenesis, and memory. In Alzheimer's disease, disruption of the BDNF/TrkB signaling axis is associated with synaptic dysfunction, neuronal loss, and cognitive decline (Alqahtani et al. 2025). CTSB is another candidate mediator of muscle–brain crosstalk and is associated with hippocampus‐dependent memory (Chow et al. 2022). In a recent study on mice, treadmill running increased CTSB levels in the skeletal muscle, which was released in extracellular vesicles, followed by CTSB delivery to the hippocampus. Wild‐type mice exhibited increased hippocampal neurogenesis and better memory. APP/PS1 mice showed lower amyloid‐beta deposition, less neurofibrillary degeneration and neuroinflammation, and better cognitive performance. When CTSB was knocked down in the muscle, the exercise effect weakened; when CTSB was overexpressed, it was strengthened (Zhou et al. 2026).

**FIGURE 1:**
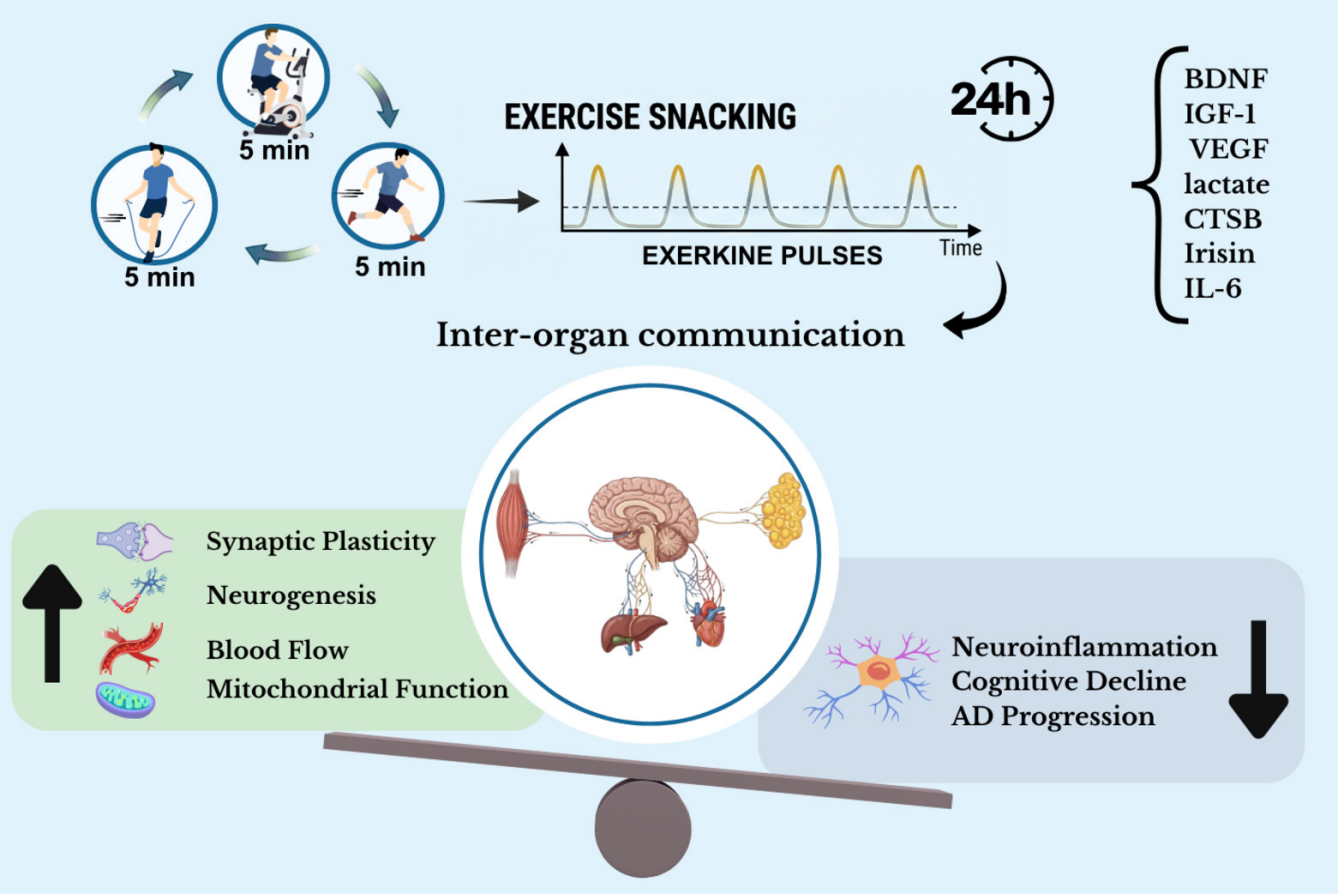
Proposed repeated exercise‐pulse model for exercise snacking in Alzheimer's disease. Brief bouts distributed throughout the day are hypothesized to generate repeated exercise‐induced signaling events within 24 h. Because candidate mediators differ in their kinetics and transport mechanisms, the model does not assume uniform pulses across molecules. Candidate signals include BDNF, IGF‐1, VEGF, lactate, CTSB, irisin, and IL‐6. The model places skeletal muscle within a wider organ‐to‐brain signaling network that also includes the adipose tissue, liver, heart, and brain. Repeated activation of this network is hypothesized to affect synaptic plasticity, neurogenesis, cerebral blood flow, mitochondrial function, neuroinflammation, cognition, and Alzheimer's disease‐related pathology.

Over the past few years, our group has shown in rodent models that high‐intensity ES can produce greater adaptations in selected cardiometabolic and brain‐related outcomes than single‐session protocols matched for total volume and intensity. In rodents, several short high‐intensity sessions performed throughout the day improved insulin sensitivity and reduced visceral fat more effectively than single longer daily sessions (Mendes et al. 2022). We subsequently examined whether this temporal pattern also influenced the aging of the hippocampus. Notably, in aged rats, three daily 5‐min HIIT sessions separated by 4 h induced hippocampal adaptations that were not fully reproduced by single time‐matched 15‐min sessions. Both protocols improved aerobic fitness and spatial memory in the rats. However, only the ES protocol reduced anxiety‐ and depression‐like behaviors, increased hippocampal CaMKII, and resulted in higher hippocampal BDNF levels than the single‐session HIIT (Magalhães et al. 2024). These findings derive from high‐intensity paradigms and should not be generalized to all forms of ES. Whether low‐to‐moderate‐intensity exercise snacks can generate biologically meaningful exerkine responses remains to be determined. We therefore propose that the differences observed with high‐intensity ES may arise, at least in part, from repeated exerkine pulses across the day, which could reactivate short‐lived molecular signaling windows more frequently than a single daily exercise session.

Testing this idea requires three distinct lines of work. The first is mechanistic and belongs in animal models of Alzheimer's disease: does ES generate a brain molecular signature that a single volume‐matched session does not, and does that signature translate into measurable changes in hippocampal plasticity, neuroinflammation, mitochondrial function, cerebrovascular regulation, and amyloid or tau pathology? Human studies pose a different challenge. Peripheral exerkines are straightforward to quantify, but a rise in the blood reveals little about the brain unless the two are measured in parallel; serial sampling must therefore be coupled to brain‐related outcomes. A more elementary question precedes all of this. Can people with cognitive impairment or Alzheimer's disease perform repeated bouts safely, and what intensity and dose are required to elicit measurable exerkine responses?

For now, exercise snacking is best viewed as a promising non‐pharmacological strategy for the prevention and management of Alzheimer's disease, not as an established intervention. Its appeal is temporal. Spreading the same exercise across the day may expose the brain to repeated exerkine pulses, a rhythm of molecular signaling that a single daily session may not reproduce. That is what future work should test: Does the “exerkine advantage” hypothesis apply to the Alzheimer's brain?

## Author Contributions

**Leiriel Cristina dos Reis Vieira:** conceptualization. **Ramona Ramalho de Souza Pereira:** visualization. **Ricardo A. L. De Sousa:** conceptualization, writing – original draft. **Marco Fabrício Dias‐Peixoto:** writing – review and editing. **Joyce Mirlane Moreira Costa:** writing – original draft. **Caíque Olegário Diniz e Magalhães:** conceptualization, writing – review and editing.

## Funding

M.F.D.‐P. is supported by the Conselho Nacional de Desenvolvimento Científico e Tecnológico (CNPq; grant no. 304455/2026‐1), FAPEMIG (grant no. APQ‐01160‐26) and Coordenação de Aperfeiçoamento de Pessoal de Nível Superior (Finance Code 001). Open access funding was provided by CAPES through the CAPES/Wiley agreement.

## Conflicts of Interest

The authors declare no conflicts of interest.

## Data Availability

Data sharing not applicable to this article as no datasets were generated or analysed during the current study.
